# Riluzole Enhances the Response of Human Nasopharyngeal Carcinoma Cells to Ionizing Radiation via ATM/P53 Signalling Pathway

**DOI:** 10.7150/jca.41217

**Published:** 2020-03-04

**Authors:** Lu Sun, Cheng Wu, Jun Ming, Xin Nie, Ergang Guo, Wei Zhang, Guoqing Hu

**Affiliations:** Department of Oncology, Tongji Hospital, Tongji Medical College, Huazhong University of Science and Technology, Wuhan, Hubei Province, China

**Keywords:** riluzole, NPC, cell-cycle arrest, radiotherapy, nasopharyngeal carcinoma

## Abstract

Riluzole is approved by the FDA as an amyotrophic lateral sclerosis (ALS) drug. Previous studies showed that treatment with riluzole suppressed the proliferation of many cancer cells. However, little is known about its effects on nasopharyngeal carcinoma (NPC) and its molecular mode of action. In this study, we determined the effect of riluzole on apoptosis, cell cycle, migration, and invasion in NPC cell lines and investigated its mechanism at the molecular level. By using the human NPC cell lines CNE1, CNE2, and HNE1, we revealed that riluzole effectively inhibited viability of the NPC cell lines in dose- and time-dependent manners. Furthermore, riluzole dose-dependently induced apoptosis and G2/M cell cycle arrest in the NPC cell lines. After combination with radiotherapy (RT), greater cytotoxicity was achieved than with riluzole or RT alone *in vitro* and* vivo*. This was associated with the activation of ataxia telangiectasia mutated (ATM) and phosphoinositide p53 pathways. P53 silencing reduced cell reactiveness to riluzole therapy. These observations demonstrate that the riluzole-activated ATM/P53 pathway is directly involved in radiation-induced apoptosis of NPC cells. Given the acceptable side effect, combining of riluzole and radiotherapy is promising in NPC treatment.

## Introduction

Nasopharyngeal carcinoma is a malignant tumor originating from the epithelial lining of the nasopharynx. In southern China, its incidence is 25-50 per 100,000 people, a value much higher than other areas 1. The occurrence of NPC may be attributed to Epstein-Barr viral infection, genetic susceptibility, and geographic distribution 2. To date, radiotherapy remains the preferred treatment to cure non- disseminated disease. Platinum/gemcitabine-based chemotherapy is required to improve the rate of survival at all stages of metastatic disease 3. Although radiotherapy is a powerful method for NPC, radiation-induced stress response and resistant gene expression could seriously undermine its effectiveness. Radioresistance may cause therapy failure and distant metastasis of NPC 4,5. Therefore, further exploration of the mechanism of chemoradioresistance and the discovery of new drugs to improve therapy-resistance may provide a foundation for improvements the survival rate and the aid in the establishment of a new treatment strategy for NPC.

Riluzole/Rilutek®(2-amino-6-trifluoro-methobenzothiazole) was approved by the FDA for the treatment of ALS 6. Over the last decade, large-scale research has proven that riluzole plays a role in the treatment of malignant tumors. To add, it was identified to possess antineoplastic properties against melanoma, glioma, breast, and prostate cancer by decreasing the release of glutamate 7-12. Some studies with riluzole are close to being successfully applied in the clinic and increasing interest has been shown for its use as a treatment for malignant tumors. There is some evidence that riluzole can increase the response of cancer to different anti-tumor treatments, including chemotherapy and radiotherapy 13-16.

DNA damage is the key response triggered by irradiation. DNA damage responses and repairs (DDRs) are the main processes in the determination of cell's fate by controlling DNA repair, cell cycle arrest 17. The ATM/Chk2/p53 pathway plays a critical role in the regulatory network of DDRs 18. The ataxia telangiectasia mutated (ATM) is rapidly activated when DNA damage occurs. When the damage is unrepaired, the downstream apoptotic pathway and cell cycle checkpoint can be activated by the cascade system and lead to cell growth arrest or death. Disorder in the ATM/Chk2/P53 pathway is closely related to the radiosensitivity of many tumors 19, 20. A previous study proved that riluzole could induce mitotic arrest and be accumulated in the G2/M phase in breast cancer cell lines via the ATM/p53 pathway 21. Hence, we supposed that riluzole might regulate the radiosensitivity of NPC cell lines through the ATM/CHK2/P53 pathway.

Consequently, we carried out this research to explore the impact of riluzole on apoptosis, cell cycle, migration, and invasion in NPC cell line, and investigate the mechanism at the molecular level.

## Materials and Methods

### Reagents

The primary antibodies, p-p53, p-chk2, chk2, γ-h2ax, p-γ-h2ax, monoclonal rabbit antibodies, P53 and GAPDH mouse antibodies were got from cell signalling technology (Danvers, MA). Rabbit anti-human antibodies against p21, Bax, bcl2, cyclin B, and caspase 3 were obtained from proteolytic enzyme technology (Wuhan, China). Secondary antibody was purchased from proteineach technology (Wuhan, China). Red fluorescent antibody, Cy3 goat anti-rabbit and anti-mouse secondary antibody were purchased from Invitrogen (Carlsbad, CA). The matrix gel (356234) was obtained from Corning (Corning, NY).

### Cell lines culture

CNE1, CNE2 and HNE1, human NPC cell lines, were purchased from the Cancer Research Institute of Central South University (Changsha, China). All were maintained in RPMI-1640 medium (Hyclone, USA) plus 10% FBS (Gibco, USA) at 37℃ with 5% CO_2_.

### Western immunoblots

Total protein was extracted using RIPA buffer (Biotime, China) containing protease inhibitor phenylmethylsulphonyl fluoride (PMSF) and phosphatase inhibitors for further analysis. Cell lysates were separated on sodium dodecyl sulphate-polyacrylamide gels and imprinted onto PVDF membranes. Then incubated with primary antibodies at 4°C for 12 h. Membranes were subsequently incubated with relevant secondary antibodies for 2 h at 37 °C. Images were captured by SynGene G (Alpha Metrix Biotech, Hesse, Germany).

### Colony formation assay

Human NPC cells were counted. Cell suspensions of different concentrations were seeded in 6 plate-dishes. Cells were treatmented with different doses of radiotherapy ranging from 0 to 10 Gy. Then 25 μM riluzole was added for 48 h after RT. Control group treated with DMSO for 48 h. After 2 weeks, ice-cold 4% paraformaldehyde was added to cells for fixing it. Cells were stained with 1% crystal violet. Images were captured by microscope. Colonies (more than 50 cells) were calculated by ImageJ.

### Cell Cycle Analysis

A total of 2*10^5^ cells were plated in 6-well plates for 24 h and treated with riluzole. Control group was treated with DMSO. A single-cell suspension was obtained when cells were grown to 70-80% confluence. After washing with cold PBS 3 times, cells were suspended with 70% ethylalcohol at -4 °C for 12 h. Finally, the cells were stained with Annexin V/ propidium iodide (PI) for 1-2 h at 37 °C. The flow cytometry assay was analyzed using a flow cytometer.

### Cell apoptosis analysis

Cells were counted and plated in 6-well plates and cultured for 24 h. Cells were treated with 25 μm riluzole and 6 Gy RT alone or combination for 48 h, Annexin V-FITC apoptosis detection kit was used to evaluate apoptotic cell death according to the manufacturer's instructions. Flow cytometric analysis was performed with a flow cytometer.

### Plasmid construction and cell transfection

TP53-siRNA plasmid (GV102) and a control siRNA were synthesized by GeneChem Co., Ltd (Shanghai, China). A suspension of CNE2 cells was prepared and diluted to 3×10^4^/mL. Subsequently, 500 μL was plated into a 24-well plate and cultured for 24 h. Vector plasmid and siTP53 plasmid were transfected into CNE2 cells with Lipofectamine 3000 Transfection Reagent (Invitrogen, USA) following the manufacturer's protocal. TP53 stable knockdown CNE2 cells were selected with G418.

### Immunohistochemistry

Consecutive sections (3‐5 μm thick) were dewaxed in xylene and rehydrated through graded alcohol. Ten millimolar citrate buffer was used for antigen retrieval then heated for 10 min. Slides were cooled and incubated with normal goat serum for 30 min at 37℃ to block nonspecific staining. The primary antibodies were incubated at 4 °C overnight. After incubation with the corresponding secondary antibodies for 1 h at 37℃, followed by counterstaining with Mayer hematoxylin.

### Cellular invasion assay

CNE2 cells were seeded in plates without FBS for a 12 h starvation. The transwell technique was applied to assay the ability of invasion and migration of cancer cells. A total of 1*10^5^ CNE2 cells were suspended with FBS-free medium and plated in the upper chamber. FBS-free medium was added in the bottom chamber. After riluzole incubation for 48 h, Cells in the upper chamber were fixed with iced paraformaldehyde, followed by dyeing with crystal violet. Images were captured with a microscope (five fields per chamber). Each assay was repeated 3 times separately.

### Wound healing scratch assay

CNE2 cells were seeded at 2×10^5^ cells per well in 6-well plates and grown as monolayers in triplicate. Cells were starved overnight, and a scratch was induced by 10 μL plastic pipette tip. Cells were cultured with or without riluzole. The wound was examined and photographed with a photomicroscope at different time points. Cell migration was evaluated by Image J.

### Murine xenograft model

A total of 5*10^4^ CNE2 cells were injected into the flanks of female nude mice. Mice were randomly divided into 4 groups when the tumors reached approximately 100-150 mm^3^. Group 1 was treated with DMSO as vehicle(Control) every other days, group 2 was treated with riluzole (10 mg/kg) by intraperitoneal injection(Riluzole) every other days. group 3 was treated with 8 Gy radiotherapy (RT) at first day and followed intraperitoneal injection (DMSO) every other days, and group 4 treated with 8Gy radiation at first day + 10mg/kg riluzole (RT+riluzole) every other days. The length and width of tumors was measured with vernier calipers every there days. When tumor volume reached 2000 mm^3^, mice were sacrificed by cervical dislocation. Tumors were rapidly dissected, and half were snap-frozen to -180℃ and while the other half was fixed with 4% polyformaldehyde for immunohistochemical study.

### γ-H2AX immunofluorescence

Cells were treated with RT or riluzole after being plated on the slide. After fixed and washed, cells were permeabilized with 0.5% Triton X-100/PBS for 30 min at 37 ℃. After blocking with 5% goat serum, cells were incubated with primary antibodies, γ-H2AX , for 12 h at 4 ℃. The cy3-conjugated secondary antibody was added to cell slide for 2 h at 37 ℃, then stained with DAPI, a DNA-specific probe. Cells were mounted with laser scanning confocal microscopy (Zeiss, Germany) and images were acquired. Picture processing was analyzed by ImageJ 1.8.0.

### Statistical analyses

Numerical data were analyzed by GraphPad Prism (v.7.0). Two‐sample student t-tests or ANOVA tests followed by a post-hoc Bonferroni test were used for statistical analyses. Unless otherwise indicated, all numerical results are expressed as mean ± SEM. Comparisons were made between treated and either untreated or vehicle-treated samples. P < 0.05 is considered statistically significant.

## Results

### Riluzole inhibits tumor growth and metastasis *in vitro*

**Figure 1:**
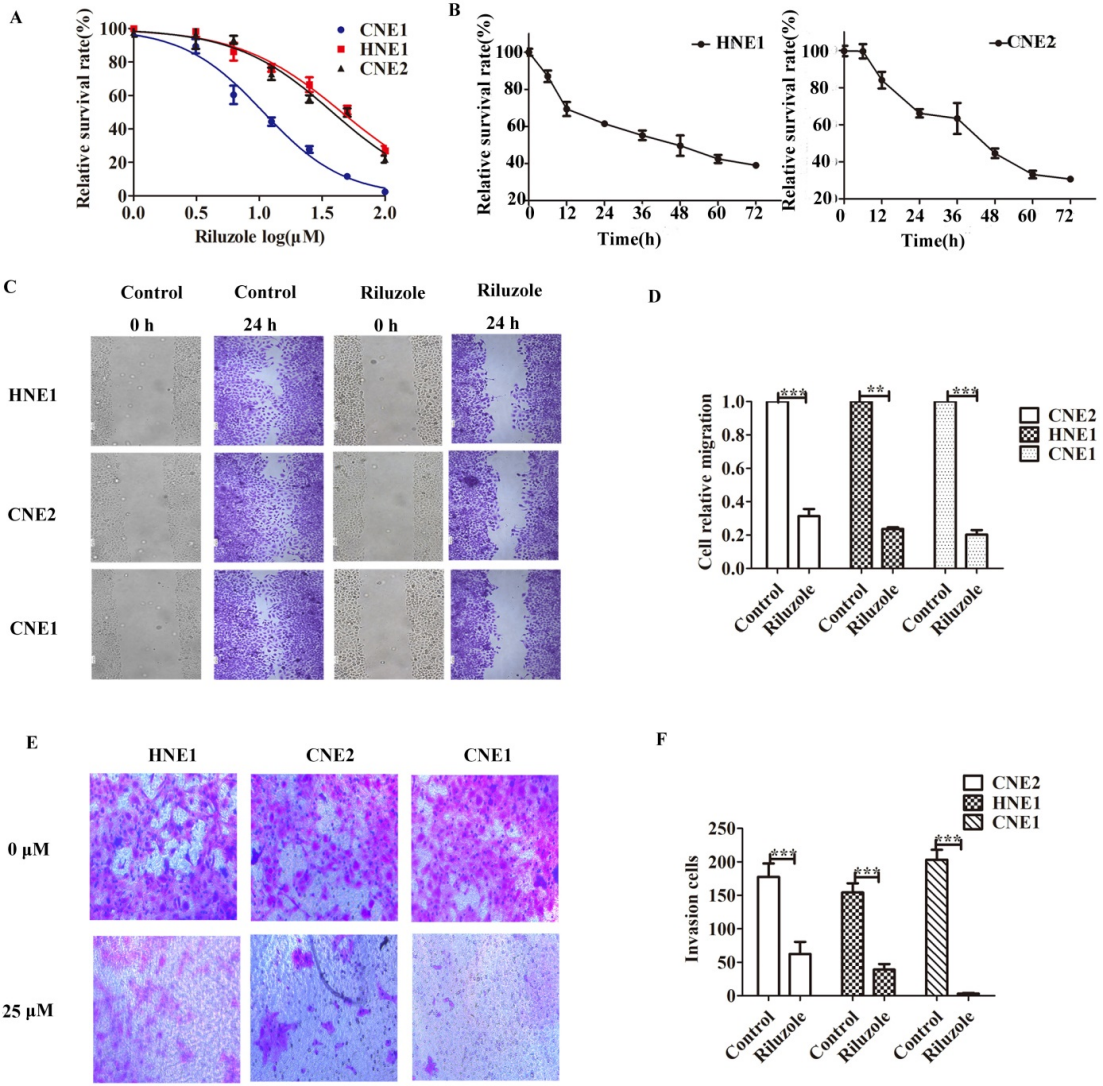
Riluzole inhibits cell proliferation, migration and invasion of various NPC cells. **A** CCK8 cell viability/proliferation assays were performed with the HNE1, CNE2, and CNE1 cell lines. **B** CNE2 and HNE1 were treated with control (DMSO) or riluzole (25 μM). Absorbance was calculated at each time point. **C** The migration of NPC cells was tested by scratch assay. Representative images of the scratch were captured at 0 h and 24 h. **D** Riluzole can inhibit the metastasis of NPC cell lines. Students t-test were used. *** P < 0.001. ** P < 0.01. **E** Riluzole inhibits the ability of cell invasion. Images were captured at 24 h.** F** The number of cells invading the down chamber was counted and analyzed by GraphPad. Students t-test were used. *** P < 0.001 vs. the control groups.

First, several established NPC cell lines were incubated with riluzole to determine its effect on cellular proliferation. CCK8 analysis was performed. We observed that the apoptotic rate of cells reached 50% when CNE2 and HNE1 treated with 25 μM riluzole and CNE1 treated with 10 μM riluzole (Fig. 1A). Then CNE2 and HNE1 were exposed to 25 μM riluzole for different times (Fig. 1B). Further determine the effects of riluzole on NPC cell migration and invasion, wound-healing and transwell invasion technology were employed. For the riluzole treated cells, the number of cells migrated into the wound was drastically lower than that of the control (Fig. 1C). This finding reveals that treatment with riluzole (25 μM, 24 h) inhibits the migration of CNE1, CNE2, and HNE1 cells (Fig. 1D). Upon riluzole treatment, the average number of NPC cells invade to the lower chamber significantly decreased (3.333±0.88, 62.33± 18.27, and 39.00±8.38, respectively) compared to control cells (203.3 ±14.77, 177.7 ±19.88, and 154.3±13.69, respectively) (Fig. 1E, F). Altogether, Such finding suggest that riluzole inhibits the proliferation of the NPC cell lines in a time and dose dependent manner. The ability of migration, invasion and viability of NPC cells also could be reduced by riluzole.

### Riluzole induced G2/M phase arrest in NPC cell lines

**Figure 2:**
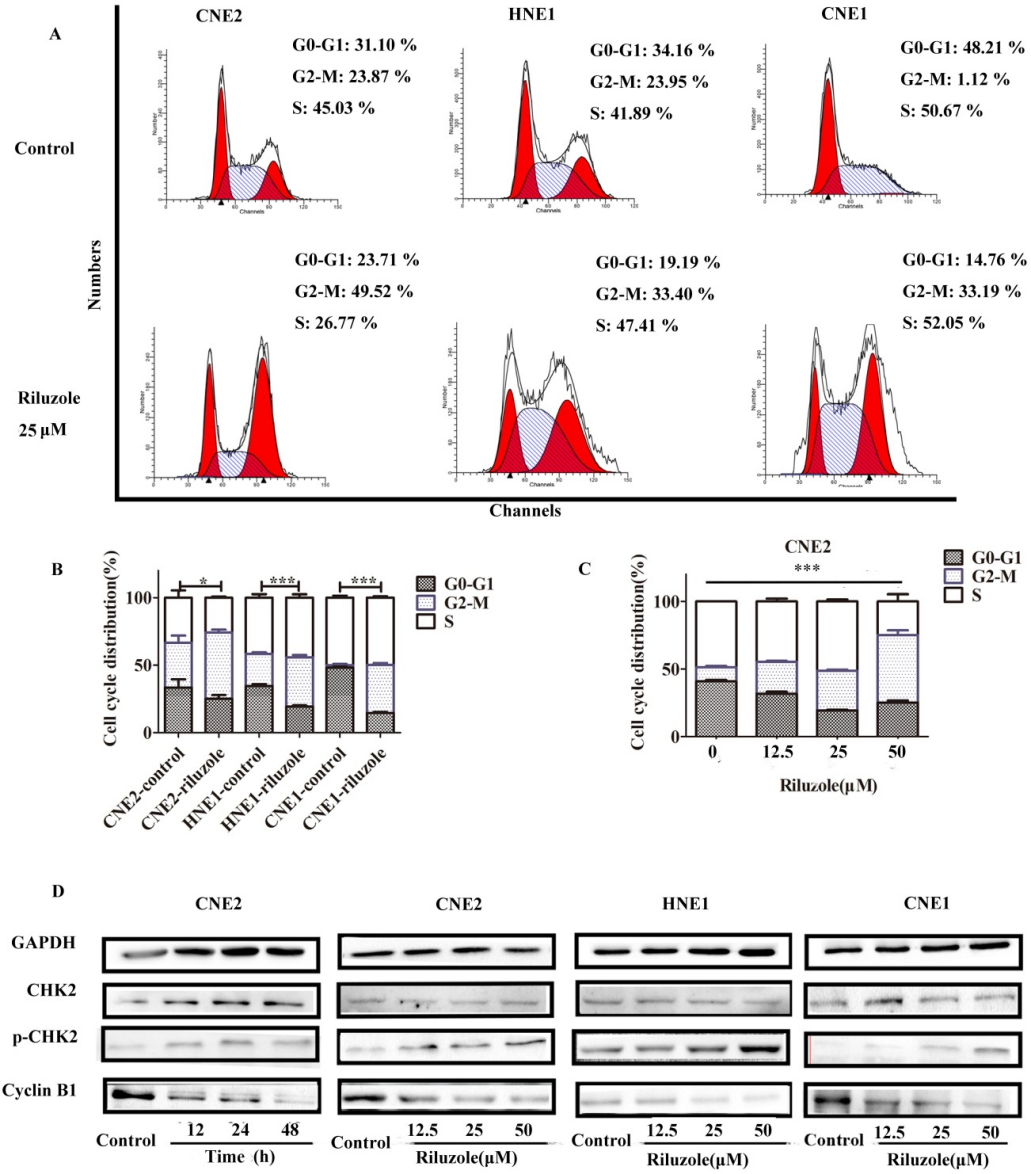
Cell cycle distribution in NPC cells after treatment with riluzole. **A, B** Riluzole increased the number of cells in the G2/M-phase for CNE2, CNE1, and HNE1 cells. *, P < 0.05 vs. the control groups; ***, P < 0.001 vs. the control groups. Students t-test were used **C** Riluzole induced G2/M cell cycle arrest in a dose-dependent manner. Data were presentes as mean±SEM. One-way ANOVA were used. ***, p<0.001. **D** Mitotic cell cycle proteins were detected by western blot. Results are representative of three experiments.

To explore the intrinsic mechanism whereby riluzole inhibits cell growth, we evaluated its effect on cell cycle progression in the NPC cell lines. As presented in Fig. 2A and B, after 48 hours treatment, cells exposed to riluzole demonstrated a statistically significant two-fold shift to the G2-M phase compared to control cells. Given the effect of riluzole on the cell cycle, we explored its effect on the levels of proteins involved in cell cycle regulation. Cells treated with riluzole had a time and dose-dependent decrease in the expression of Cyclin B1, which was accompanied by an increase in CHK2 phosphorylation in the cell lines (Fig. 2D). The results reveal that riluzole therapy could induce G2/M synchronization in NPC cells. It is widely accepted that cells in the G2-M phase are more sensitive to radiation therapy (RT) 22. This finding could support our hypothesis that riluzole may enhance the radiosensitivity of NPC cells.

### Riluzole Enhances Radiosensitivity in NPC Cell Lines

**Figure 3:**
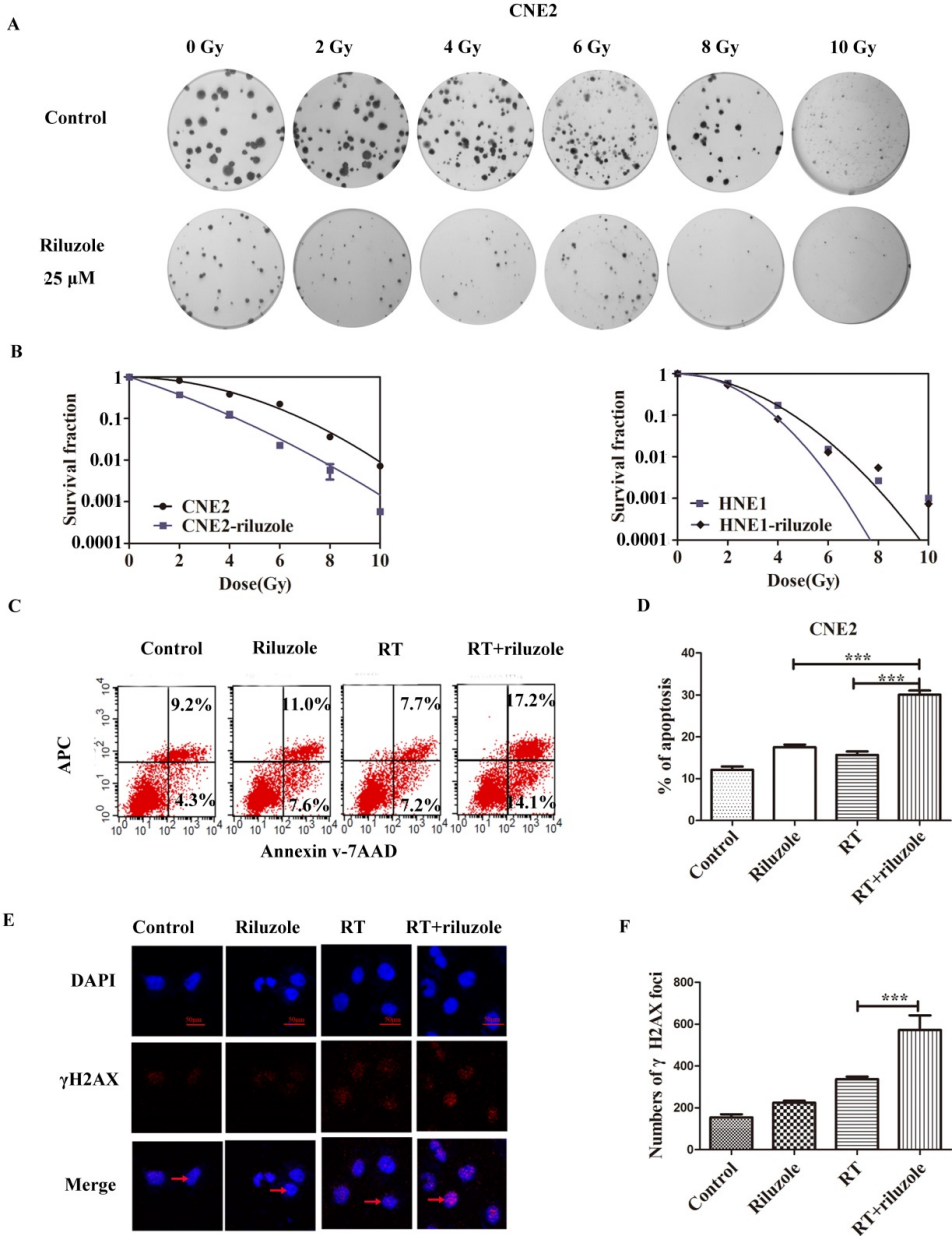
Riluzole and RT enhance cell apoptosis. **A, B** Colony assay was performed with HNE1 and CNE2 cells in the presence of 25 μM riluzole, and their totals were reported relative to DMSO-treated cells, demonstrating fewer colonies with the combination of riluzole and RT. **C, D** Riluzole can increase the rate of apoptosis in radiation-treated cells. Data from multiple independent experiments are presented as mean±SEM. One-way ANOVA with Bonferonni's multiple comparison test was used. ***, P < 0.001. **E, F** Immunofluorescence stains for DAPI (blue), γ-H2AX (red), or merged. The arrow points to the γ-H2AX which was stained in nucleus. Cell nucleus was stained with DAPI. Bar, 50 μm. One-way ANOVA with Bonferonni's multiple comparison test was used to calculate statistics. RT vs. RT+riluzole ***, P < 0.001.

To investigate the effect of riluzole on the response of cells to radiotherapy, colony formation assay was performed by treating CNE2 and HNE1 with RT, followed with 25 μM riluzole incubation for 48 h or not (Fig. 3A). The results are summarized in Fig. 3B. As expected, the combination of RT with riluzole significantly reduced the survival fraction in NPC cells. Besides, an apparent abrogation of the initial “shoulder” was found, especially in CNE2, in the cell survival curve. CNE2 had a higher survival rate and an increase in SF_2_ value by comparation with the riluzole+RT group (0.54 and 0.42 respectively, Table 1; Fig. 3A and 3B). Change in SER for the riluzole+RT group was 1.27-fold higher that of the control (Table 1), indicating that riluzole can induce radiosensitivity. Its impact on HNE1 was also statistically significant. As apoptosis is a key cellular response to therapy, the apoptotic rate of different groups was detected using flow cytometry methods. It is evident that the combination of riluzole and RT significantly increased the apoptosis of NPC cell lines (Fig. 3C, D). The apoptotic rate of the control, riluzole, RT, and combination groups were 12.10±0.8110, 15.70±0.8220, 17.52±0.6343, and 30.10±0.9953%, respectively (Fig. 3C). DNA double strand break (DSB) is the main change after radiotherapy. γ‐H2AX, a marker of DSB, was verified by immunofluorescence assay. After different treatments, the number of γH2AX foci was counted and detected by confocal microscopy. The level of DSBs was intense in the combination group relative to the control group (Fig. 3E, 3F). Such consequences support the notion that riluzole could increase DNA damage and the radiosensitivity of NPC cells.

### Riluzole induces radiosensitivity via the p53 pathway

**Figure 4:**
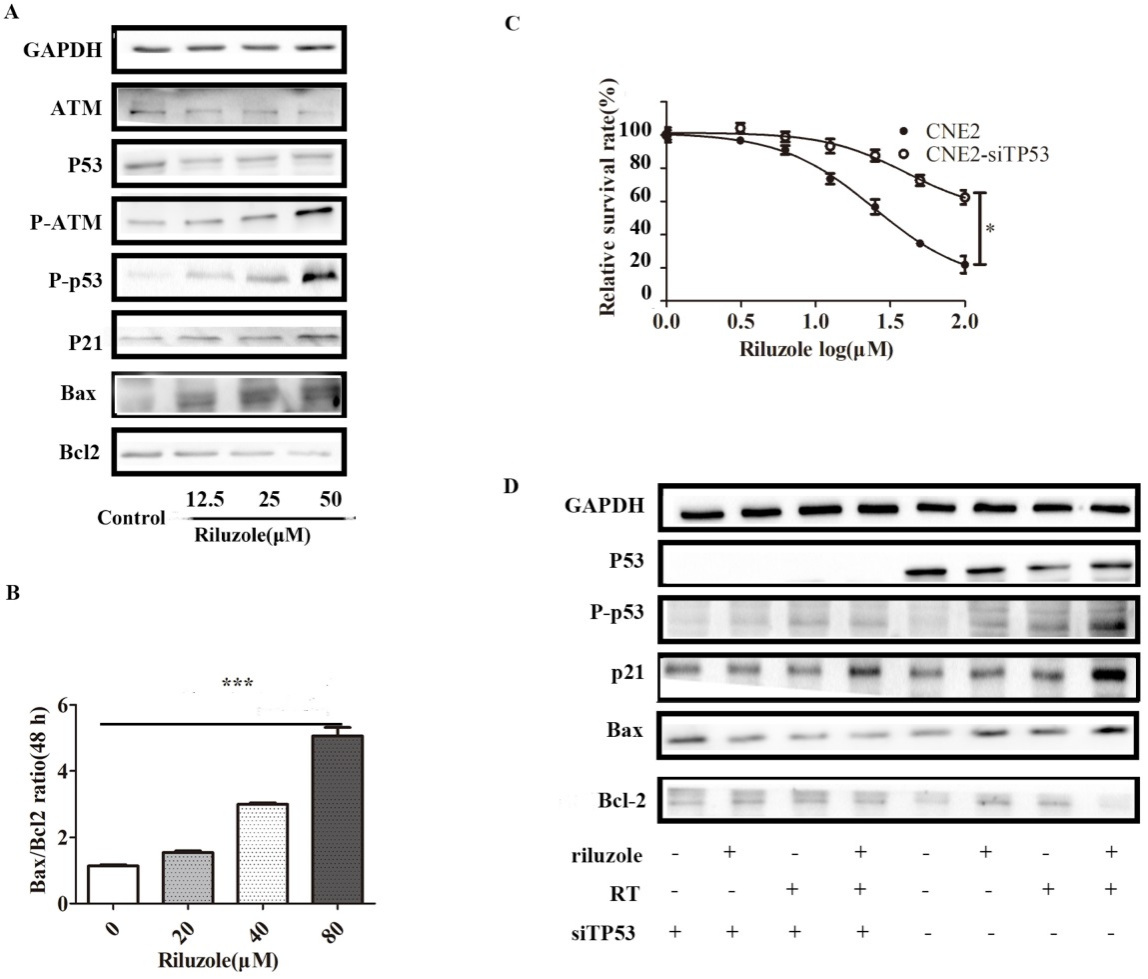
Riluzole enhances radiosensitivity through the activation of the p53 pathway. **A** Cells were treated with riluzole for 48 h, and then collected. Proteins were isolated and detected by western blot analysis.** B** Combination group showed an increase in Bax/Bcl‐2 ratio compared to other groups. Data presented as mean±SEM. Differences between indicated groups were measured by one-way ANOVA test. ***, P < 0.001. **C** The proliferation rate of the CNE2-siTP53 cell lines treated with riluzole was significantly higher than that of the control cells. Differences between two groups were analyzed by students t test. * , P < 0.05. **D** Cells were treated with riluzole and/or RT. After 48 h of treatment, cell proteins were isolated and detected by western blot analysis.

As riluzole treatment reduced the proliferation, invasion, migration and viability of NPC cell lines, we sought to explore the underlying molecular mechanism for these activities. Considering that riluzole induces G2/M phase arrest, we speculated that riluzole might activate the G2/M regulators. Previous studies reported that p53 controls cell cycle progression and induces apoptosis 23,24. To determine whether cell cycle arrest is triggered by the activation of ATM/p53, the levels of ATM/p53, phosphorylated ATM/p53 and its downstream targets, P21, Bax and Bcl-2 were investigated by Western Blot analysis. After cells were incubated with different concentrations of riluzole for 48 h, the western blots showed that riluzole increased the activation of p53 and ATM in a dose-dependent manner. Consistently, The downstream pathway was significantly elevated after treatment. These results suggest that riluzole can activate the ATM/p53 pathway (Fig. 4A). In addition, the Bax/Bcl‐2 ratio, the marker of apoptosis, is increased when CNE2 cells were exposed to riluzole (Fig. 4B). Then, P53 was silenced in CNE2 by siTP53 plasmid. CNE2 and CNE2-siTP53 were treated with riluzole at different dose. Interestingly, the apoptosis rate of p53 knockdown cells treated with riluzole was decreased relative to that of control cells (Fig. 4C). Protein abundance of the cell cycle regulators and apoptosis-related proteins remained unchanged when p53 was silenced (Fig. 4D). Collectively, these results indicate that riluzole activated p53 and induced the expression of p21, finally triggering apoptosis and cell cycle arrest. These findings suggests that the riluzole mediated apoptosis and cell cycle arrest in a p53 dependent way.

### Combination of riluzole and radiation therapy is an effective treatment for NPC in vivo

**Figure 5:**
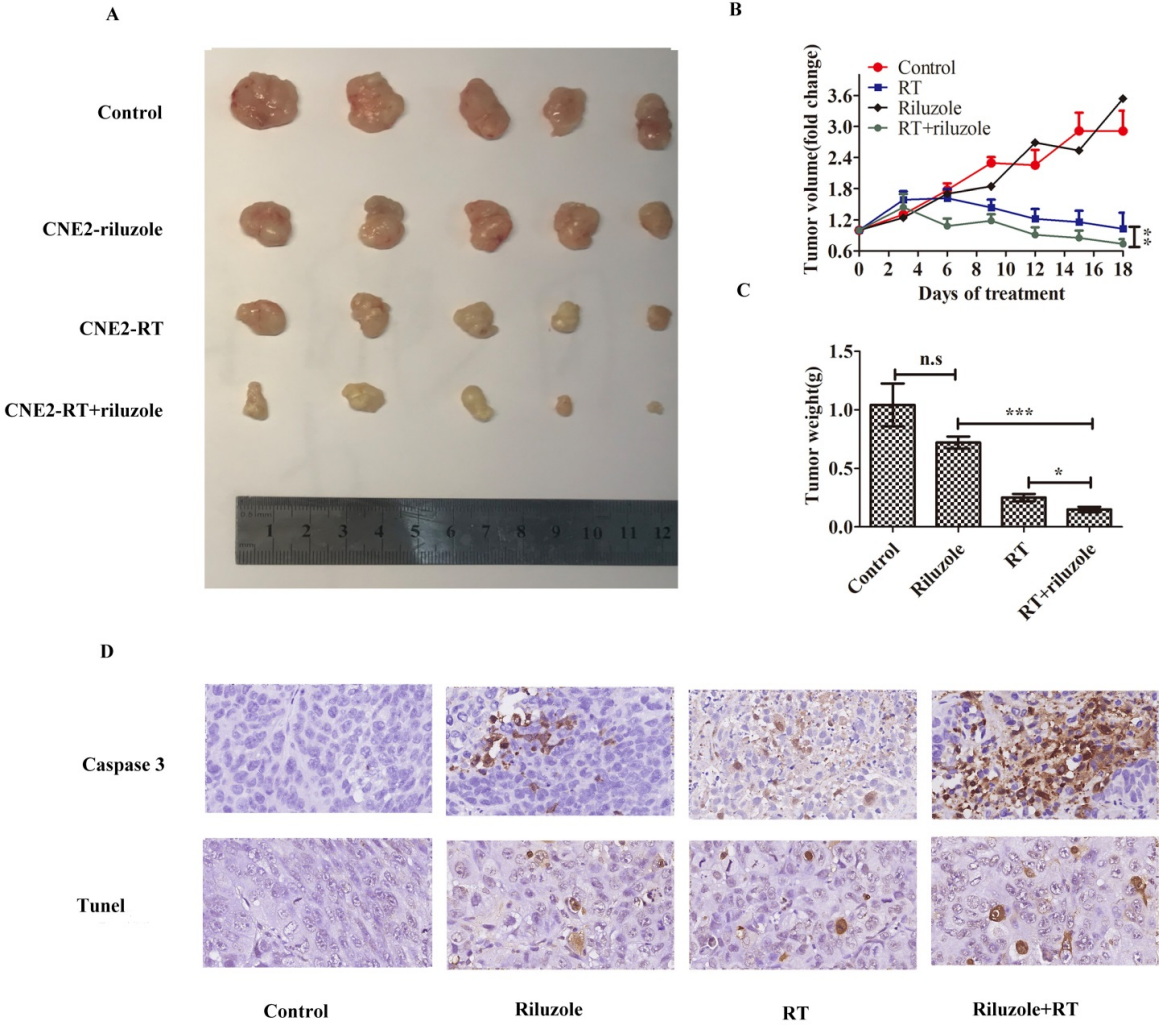
Riluzole affects the radiosensitivity of NPC *in vivo*. **A** Representative tumor xenografts of each group. **B** The volumes of tumor in the riluzole+RT group were significantly smaller than others. Data shown are mean±SEM. Differences between indicated groups were measured by one-way ANOVA test after by bonferonni's multiple comparison test *: p<0.05. **C** The weight of tumor in the riluzole+RT group were ligter than others. Data were shown as mean±SEM. Differences between indicated groups were measured by one-way ANOVA test with bonferonni's multiple comparison test. ***, P < 0.001; *: p < 0.05.** D** Tunel and cleaved caspase 3 were detected. Images were captured and analyzed (800×).

Human NPC cells cultured* in vitro* were more sensitive to irradiation in the presence of riluzole. Hence, we sought to establish xenograft experiments to determine whether this observation could be confirmed *in vivo*. xenografts were established in female (n = 20) mice using CNE2 cells. Subsequently, mice were divided into the groups: control (DMSO), riluzole (10 mg/kg), RT (8Gy), or combination of riluzole (10 mg/kg) and RT (8 Gy). DMSO and riluzole were administered every two days via intraperitoneal injection and all mice were sacrificed after 18 days due to tumor burden in the control group. Tumor volume was measured every three days to detect whether riluzole could enhance the response of xenograft tumors to the ionizing radiation and arrest or delay their growth. Our results demonstrate that irradiation alone or combination with riluzole resulted in significantly smaller tumors than riluzole alone or vehicle-treated control xenografts after 6 days. The change was more evident in the combination group. However, there was no difference between riluzole alone and control groups (Fig. 5A, B, C). Cleaved-caspase 3 and Tunel were measured by immuneo-histochemistry to reveal the level of apoptosis (Fig. 5D). As a result, an increase was found in the number of apoptotic tumor cells in the riluzole+ RT group than in the RT or riluzole alone and the control groups (p<0.001). It means that riluzole can enhance the respondence of NPC to RT *in vivo*.

## Discussion

Although NPC is highly sensitive to ionizing radiation, in many cases, radioresistance inevitably occurs 25. New substances are needed to enhance the radiosensitivity of tumors and minimize its side effects on normal tissues. Riluzole has been widely reported as a promising anticancer agent in cancer prevention and therapy. In a previous study, Hwa Jin Lee and Brian A. Wall found that treatment with riluzole augmented DNA double-strand breaks (DSBs) and G2/M phase arrest 26. These findings demonstrate that riluzole may act as a radiosensitizer in human tumor by inducing G2/M synchronization and caspase-dependent apoptosis 15. However, the underlying mechanisms remain largely unknown. A further understanding of its effect on human NPC proliferation and the development of combination treatment will result in multiple applications that extend far beyond the traditional roles, ultimately benefiting many people.

We designed some related experiments to validate our hypothesis. In this study, treatment with riluzole had effects on proliferation, cell cycle, migration, invasion, and apoptosis in NPC cell lines. Riluzole-induced apoptosis was variable and cell line-specific. A highly differentiated NPC cell line, CNE1, was more sensitive to riluzole than the poorly differentiated cell line. Genomic variability between NPC cell lines may be responsible for the susceptibility of riluzole. Although riluzole has an antineoplastic effect on poorly differentiated cell lines, we haven't designed protocols elucidate its cause in this study. Nonetheless, we demonstrated that riluzole could alter the expression of proteins involved in cell cycle regulation in time and dose-dependent manners. Consistent with the previous research results, cells treated with riluzole were accumulated in the G2/M phase in NPC cell lines. It is well known that cancer cells in the G2/M phases will be particularly sensitive to ionizing radiation. Unrepaired DNA damage induced by radiation, usually DSBs, will lead to mitotic catastrophe and cell death 27. We observed that the combination of riluzole and radiotherapy positively correlated with the apoptosis after irradiation *in vitro*.

In humans, cell division is the basis for proliferation, growth, and repair of damage. Hence, a the precise checkpoint is required to ensure reliable copy inheritance to the next generations. In the entire cell cycle, cells contain monitors to induce cell cycle arrest, even apoptosis. To add, these monitors can identify unintegrity and mistakes in the genome. The G1/S and G2/M checkpoints are especially relevant to cancer treatment. G1/S checkpoint causes cells to pause before embarking on the S phase when DNA is damaged. G2/M checkpoint can repair DNA damage and induce apoptosis upon being unrepaired. Accumulating evidence has confirmed that cell cycle arrest may result in radiosensitivity 28-30. Cell cycle checkpoints are rapidly activated through a p53-dependent mechanism in normal cells. Tumor suppressor p53 is one of the essential proteins in the checkpoint pathways. P53 activation may result in cell cycle arrest at the G1/S and G2/M phases 31. P53 is activated by phosphorylating ATM and ATR, the main DNA damage transducers, and can be involved in various substrates phosphorylated in nuclear and extranuclear homeostasis 32. P53 can be directly phosphorylated by ATM and ATR but can also be activated indirectly via Chk2 and Chk1^33^. It can bind to the promoter of p21, causing p21 accumulation 34,35. P21, a CDK inhibitor, decreases the activity of Cdc2-cyclin B1 and drives highly damaged or stressed cells to achieve temporary cell-cycle arrest for DNA repair. When DNA damage is unresolvable, cells maintain the cytotoxic protein, BCL2 associated X(BAX), in a constitutively active state and can quickly promote programmed cell death.

In our study, we observed an increase in phosphorylated p53 levels after riluzole treatment. Hence, we conducted follow-up experiments to confirm that riluzole regulates the cell cycle and apoptosis via the p53 pathway. We achieved effective interference of p53 in the CNE2 cell line by using stable RNA interference. Such finding was also validated at the protein levels. Cells showed a decrease in cytotoxicity after riluzole exposure in the absense of p53. Further, we comfirmed that riluzole act as a radiotherapy sensitizator in *vivo*. Interestingly, the response of xenograft to riluzole monotherapy was minimal. To add, our observations do not agree with another report which proposed that breast cancer xenografts treated with riluzole had a significant decrease in tumor volume 14. However, several differences exist between this report and our work. For example, the different origins of the cells and the distinct exposure time could be responsible for their differing results. This suggests that riluzole plays a role in mediating the immune system in NPC. Riluzole can increase the survival rate of HIV-1-infected patients by inhibiting spontaneous apoptosis of T cells 36. In immune-deficient models, such as nu/nu mice, the entire immune system was absent as the T-cells were not functioned.

In conclusion, our experiments supported riluzole has anti-tumor effects and inhabits the migration and invasion on NPC cell lines which may improve prognosis of the NPC patients. The cytotoxicity of riluzole is dependent on the impact of p53 regulation on DNA damage, apoptosis, and cell cycle redistribution. Further studies on the signaling pathways and riluzole targets in NPC will be of great significance for the design of clinical trials employing this drug.

## Figures and Tables

**Table 1:** Radiobiological parameters of NPC cells exposed to radiation

Cell line	SF_2_	D_0_	Dq	N	K	SER
CNE2	0.54	2.019794	2.576534	3.581	0.4951	-
CNE2-riluzole	0.42	1.6728	0.444022	1.304	0.5978	1.207433
HNE1	0.52	1.308558	1.69709	3.658	0.7642	-
HNE1-riluzole	0.46	0.93633	1.687149	6.061	1.068	1.39754

## Abbreviations

IR: ionizing radiation
ALS: amyotrophic lateral sclerosis
NPC: nasopharyngeal carcinoma
ATM: ataxia telangiectasia mutated
RT: radiation therapy
Bcl-2: B-cell lymphoma-2
Bax: Bcl-2-associated X
TP53: tumor protein 53
DDRs: DNA damage responses and repairs
DMSO: dimethyl sulfoxide
DSBs: DNA double-strand breaks
